# Case Report: Keeping the Milan approach legacy alive? Paradox and counterparadox working therapeutically with non-suicidal self-injury

**DOI:** 10.3389/frcha.2025.1657395

**Published:** 2025-09-02

**Authors:** Ferdinando Salamino, Elisa Gusmini

**Affiliations:** ^1^ Department of Psychology, University of Northampton, Northampton, United Kingdom; ^2^ Clinical Psychology, University of Oxford, Oxford, United Kingdom; ^3^ School of Law and Social Sciences, Oxford Brookes University, Oxford, United Kingdom; ^4^ European Institute of Systemic-relational Therapies, Milan, Italy

**Keywords:** family therapy, systemic psychotherapy, child and adolescent mental health (CAMH), non-suicidal self-injury, milan approach, social constructionism, post-Milan systemic family therapy

## Abstract

Is it possible to maintain some of the precious wisdom of our ancestors, while embracing the post-modern revolution of family therapy and systemic thinking? This paper tries to offer an exploratory answer to this question. Milan Approach designed its interventions relying on the therapist’s expert position, their moral neutrality and their ability to identify, as an external observer, the “family games” that were responsible for the identified patient’s symptoms. Despite its success in offering a fresh perspective and some innovative therapeutic strategies to deal with a range of issues, including, but not limited to, eating disorders, the Milan Approach has undergone criticism, mainly due to its lack of reflexivity about social justice and elements of inequality that might have been at the foundation of problematic family dynamics. In the commendable attempt of purifying family therapy from elements of oppressive practice, post-Milan approaches have distanced themselves from their “ancestors” and showed increasing reluctance to use their tools. Particularly, counter-paradoxical interventions such as the invariable prescriptions have been progressively abandoned in favor of more collaborative tools. This paper, through the means of a clinical example, explores the usefulness of a counter-paradoxical intervention in a second-order family therapy, embracing a social-constructionist perspective while maintaining the importance of counter-paradox in allowing change. The paper discusses the underpinning principle, the delivery and the outcome of such intervention, and addresses potential criticism, indications for practice and scope for further research.

## 1 Introduction: the benefits and risks of a revolution

The Milan Approach as outlined in its foundation by Selvini Palazzoli et al. (1, 2) is a therapeutic approach deeply rooted in Gregory Bateson's (3) systemic thinking and Palo Alto's theories on human communication (4, 5).

As such, it bases its conceptualization of individual problems as an expression of a family crisis, where the “identified patient” may serve the function of a spokesperson for the family problem (2). In this respect, the identified patient is seen as trapped in a web of paradoxical communication involving the whole system, and it is the paradox itself that maintains (and gives power to) the problem.

The intervention strategy of Selvini Palazzoli's team therefore relies on a “counterparadox” (2), often taking the shape of a family ritual, which purpose is to expose, tackle and ultimately disempower the paradoxical level of family communication responsible for the problem.

Despite its success in offering a fresh perspective and some innovative therapeutic strategies to deal with a range of issues, including, but not limited to, eating disorders, the Milan Approach has undergone criticism, mainly due to its lack of reflexivity about social justice and elements of inequality that might have been at the foundation of problematic family dynamics (6). Another underdeveloped aspect of the Milan Approach, as argued by Campbell (7), was the relevance of culture and social discourse in influencing the therapist's positionality and, therefore, their hypothesizing.

In many respects, these limitations seem to stem from the same root, i.e., the early stages of development of systemic theory itself: the transition in between a first-order cybernetics (where the therapist is considered external and independent from the system being observed) and the second-order cybernetics (acknowledging the therapist as internal to the system being observed, in a circular process of mutual influence) was yet to be completed.

Still embedded in what Campbell (7) describes as a modernist approach to therapy, Milan Approach designed an intervention relying on the therapist's expert position, their moral neutrality and their ability to identify, from the position of an external observer, the “family games” (8) responsible for the identified patient's symptoms.

It was arguably the attempt to address the aforementioned criticism, together with the growing influence of social constructionism and feminist literature on family therapists across the world, that progressively led to the development of post-Milan approaches, which common ground is the acknowledgement of the therapist as internal to the system and the assumption of a not knowing, non-expert position (9), underpinned by the core principle of curiosity (10).

This sort of revolution in the field of systemic thinking presents a number of advantages, freeing the therapist from the shackles of being the sole responsible for leading the therapy toward change and empowering identified patients and families through an acknowledgement of their resources and centrality in informing their own treatment.

On a broader scale, it also aligns family therapy with the contemporary *zeitgeist*, as it promotes a democratization of knowledge and a redistribution of power not dissimilar to what we observed in other popular therapeutic modalities, such as the person-centred approach (11) and the Open Dialogue model (12).

Systemic thinking has then progressively shifted from the analysis of what function the problem has (in protecting the family homeostasis), to the exploration of the influence and power that the problem has over the family, changing dynamics, rules and roles.

The legacy of the Milan approach's divergent thinking and its strategic soul are still clearly visible in the works of Ugazio (13) and in recent developments such as the systemic-family-individual approach (14). However, it is undeniable that, as argued by Dallos and Urry (15), the vast majority of practitioners in the contemporary systemic arena seem reluctant to embrace, or even recognize, “the legitimacy of using the pioneering ideas from the first phase of systemic therapy – the notion of the function of a symptom, structural and strategic interventions, and even from the second phase – reframing and positive connotation, to take a few examples” (p. 161).

This change in paradigm is partially led by the growing attention toward the possibility of blaming parents and offending families with hypotheses and explanation that might sound harsh, which, again, is a true reflection of the cultural change in our era.

However, this commendable attempt to purify family therapy from elements of oppressive practice is also facing the risk common to all revolutions, which is to destroy, cancel and, ultimately, lose precious knowledge that constitutes the core of systemic's unique approach to human problems.

More so, as pointed out by Dallos and Urry (15), this attempt is also at risk of misinterpreting the core foundations of systemic interventions based on identifying a connection between individual symptoms and family dynamics and structures, which is that every hypothesis is generated in the territory of “as if”, a creative space where explanations are produced to empower and uncover new resources in the system, rather than a psychoeducational area of linearly connecting cause and effect.

Therefore, if we recognize the existence and relevance of this “as if” space, we can then argue that, counterparadox, strategic ordeals (16) and invariable prescriptions have always been utilized within such metaphorical space, in which therapist and client connect for a temporary, yet vital moment of co-construction, which the aforementioned “revolution” seems to have forgotten or otherwise decided to ignore. The erosion of the distinction between what is literal and what is metaphorical is at risk of being lost not only in psychotherapy, but in many areas of human life such as political and educational discourse, thus impoverishing the generative function of language.

In the hope of offering a contribution in reconsidering the importance of “as if” in family therapy, this paper aims at providing an example of how some core techniques and strategies of Milan approach, such as rituals and counterparadox, can still be effective in dealing respectfully with issues such as Non-Suicidal Self-Injury (NSSI) in the postmodern world.

## 2 Case presentation

We meet Jasmine and her family in a therapeutic centre operating in private practice in the Midlands area. The centre has a strong connection with local authorities and social workers, but families and individuals can also access the service through self-referrals, which is the case of this family. Jasmine is a 15-year old female, white British citizen. She is an only child. Her parents, Josh (53) and Ellinor (47) married 23 years ago and are still together. Ellinor has a diagnosis of major depression and is a stay-at-home mother. Josh runs a business in finance and accounting with branches both in the UK and Germany. Josh has a younger brother, Mike (47), who is currently working with him following severe mental health issues.

### 2.1 The identified patient and her presenting problem: the Fall of Jasmine

The family self-referred to our family therapy service in December 2021, due to recent episodes of self-harm (Jasmine causes herself cuts and bruises on the legs and arms, usually with scissors or other objects she might have at hand) and social withdrawal, all happened within the last eight months. Before accessing our service, Jasmine was seen by a child psychiatrist who diagnosed her with anxiety and mild depression and prescribed low dosage fluoxetine. She has also seen an individual counsellor for a few sessions, but she recently decided to quit.

During the first phone call, Ellinor discloses she is worried that Jasmine might be suicidal as she often refers to a “game” that everyone has to participate, a sort of “Squid Game” where people in power dictate roles, rules and expectations. She seems to believe that most people are unaware of partaking to the game and that, especially as a female, the game rules are strict and constraining in determining a set pathway. She has on some occasions expressed the view that the game is “rigged” against women and the desire to “get out of the game”.

Jasmine has now stopped attending school and does not leave home, if not for very short walks in the park with mom. She spends her days in her room, listening to music and reading poems. Josh and Ellinor have made every effort to hide sharp objects that Jasmine can use to hurt herself, but she seems to always find a way to self-harm.

Ellinor describes Jasmine's recent change as a “fall”: she used to be sociable, ambitious and a real hard worker, which convinced the parents to accommodate her desire to challenge herself in the very competitive environments of one of England's most prestigious, and strict, schools.

However, following a “honeymoon period” during which she seemed to integrate herself brilliantly in the new environment, she started withdrawing and, ultimately, stopped attending.

According to Ellinor, Jasmine's issues are a serious concern especially for Josh, who was extremely proud of her academic success and is now experiencing low mood and anger: “he seems to feel like she is letting us down on purpose”.

Ellinor adds that she is concerned too, but her emotions gravitate more toward anxiety and guilt, as she fears that she was not a good role model for Jasmine. Coming from a patriarchal family where women were not expected to work outside the house, she gave up on her talent as a musician and settled for low paid, temporary jobs before choosing to be a stay at home mum after Jasmine's birth: “she did not take her ambition from me, but now I fear she has taken my “quitter attitude””.

## 3 Assessment

Following the phone call, we offered a three sessions family consultation to the family.

As argued by Ugazio (17), agreeing to a consultation stage before stipulating a full therapeutic contract can offer an invaluable space for a free exploration. As it does not request the commitment of a therapeutic contract, it is an important stage in which both the therapist and the family can freely and loosely share hypotheses and ideas, engaging in a process of conjoint meaning making and assessment. Expectations, goals for therapy, different perspectives on the problem and concerns that might emerge about the therapy are thoroughly explored during the consultation.

Some possible outcomes of this consultation stage can be:

(a) an extension of the consultation itself (where the therapist and the family agree that further exploration is needed to understand whether a therapeutic intervention is needed and possible);
(b) an indication for therapy, if the therapist and the family were able to co-construct goals for therapy and a shared view on how to achieve them;
(c) a conclusion of the intervention, in the event that the therapist and the family recognized that the consultation itself was instrumental in generating potential for change. In this last case, usually, the family and the therapist agree that not all presenting problems were fully resolved, but the intervention uncovered some new resources, empowering the family so that they can find their own way of solving the problem(s). This last outcome, despite not presenting as conclusive, can be extremely useful when dealing with situations where the family can struggle to accept and/or cope with more structured, longer interventions.

This will also be the outcome of our consultation with this family, as we will detail below.

### 3.1 The pattern that connects

The Milan team led by Mara Selvini Palazzoli encouraged therapists to be extremely thorough in approaching a referral (18) not to overlook the powerful message that the referring person is sending to the family. We do not agree with the idea that the referring person is to be considered the protector of the family homeostasis, since the act of a family therapy referral might as well be underpinned and informed by different patterns and motivations. However, we concur with Ugazio (17) that themes emerging from the pattern connecting the family to the therapy are revealing of the family organization, dynamics and meaning making and need therefore to be explored. What do they seek help for? How do they define the problem and how did they try to solve it before asking for professional help? How do they choose their therapist? Who makes the call and who is in the know? All these questions are crucial in understanding the family.

This particular referral seems to be an expression of the family's culture around success and ambition. The whole narrative is about rise and fall, being determined or a quitter, power and the lack of it. Ugazio (13) suggests that the conversation in the family revolves around crucial themes, organized in a continuum defined by a limited number of semantic polarities. Among those, the “semantic of power” is identified as playing a role in informing the family conversation in families where eating disorders, but also other mental health issues such as performance anxiety, might present.

The phone call by Ellinor also highlights a concern over parenting and modelling, where Ellinor believes that she is responsible of an intergenerational transmission of surrender and failure: “I deeply fear that, because she's always seen me as a failure and a quitter, she will end up just like me”.

Lastly, the referral outlines the possibility that a blame game is taking over the conversation in the family: Josh openly blames Jasmine, Ellinor explicitly blames herself. We are left wondering whether other, more implicit and silent, elements of blame might be contributing to the problem.

With these questions in mind we invite the whole family to the first session, in order to explore the problem and gather some information on the family history.

### 3.2 Hopes, concerns and expectations

When asked about what they would like to discuss in the session, Jasmine says that she prefers her mom to introduce the topic.

Ellinor expands on what she discussed during the phone conversation. She says that Jasmine's self-harming behaviours are less concerning now, as they used to happen more often when Jasmine was regularly attending school.

We were also keen to explore the safeguarding concerns expressed by Ellinor around Jasmine's suicidal thoughts and we conducted a thorough assessment of this aspect during the first interview. It emerged that quitting the game has no connection with ideations around taking her own life, and that Jasmine never had suicidal thoughts of any sort, despite feeling sometimes depressed and withdrawn.

At the moment, the family seems to be involved in a recurring pattern: Jasmine spends most of the week at home, she reports feeling better, to which Josh and Ellinor respond by encouraging her to perhaps attend a few classes. Jasmine goes to school on Friday (for a reduced number of hours in agreement with the institute), enjoys the company of some friends, joins a morning class and spend a hour or so in the library.

When she comes back home, everything is fine and she seems to have enjoyed her time there, suggesting she is considering going back, but then the anxiety progressively builds up during the weekend (self-harm can happen then, as a way of coping with distress). As a consequence, she withdraws from classes and the pattern starts again in a couple of weeks.

Both parents express frustration at this pattern, while Jasmine remains silent. Josh states he is seeking to find a permanent solution to the problem, so that Jasmine can get “back on tracks”.

Ellinor also shares anxiety about the agreement with the school, as they gave some leeway but are putting more and more pressure on her to get Jasmine back to school. She fears that, at some point, perhaps social services might be involved.

Jasmine is open about the fact that she does not trust the therapy to be able to help. She adds that she would struggle to define what help means in this context. She is well aware that “mom and dad want me back to school”, but she is hoping that “they will open their eyes about the game” and understand that the only way to be happy is to get out of it.

Overall, the family seems to have an ambivalent perception of the therapy. On one hand, it is a last port of call before Jasmine's future is destroyed; on the other hand, whilst Ellinor seems to hold a lot of hope that it can work, both Jasmine and Josh reiterate that they are not sure what to expect and struggle to see how “just talking” can change anything.

### 3.3 Past problems, past solutions

Josh says this is not the first time that the family had to deal with mental health issues, and reports that his brother Mike was diagnosed with a psychotic onset in 2019. He was presenting paranoid ideations where he believed his family was controlling him and preventing him from having relationships by paying his love interests to push him away. He also implied that Josh was working for a government agency, which goal was to control him to make sure he stayed on the path that was designed for him.

He received treatment and is currently in remission. He lives in Germany with his partner and is currently employed at his brother's accounting and finance company.

It will later be disclosed that, although Mike is officially employed by the company, he does not really have any responsibilities there and it is more of a way for his brother to make sure he has money as he would struggle to keep a job “in the world out there”.

He currently does not speak to Josh, as he blames him for his problems, but still has contacts with Jasmine. They were very close before Mike's problem onset, and they used to spend a lot of time together discussing books and philosophical themes.

As a therapeutic team, we could not help but notice that there seems to be an echo of Mike's world view in Jasmine's current problems, as both refer to an overarching system of control directing people's life. Whilst Mike's ideas were more centred on himself as the target of the secret agenda, Jasmine seems to convey a more universal view of the problem, as the “game” controls everyone's life, especially women's.

We explored this resonance with the family and Jasmine seemed to recognise the resemblance, suggesting that “perhaps Mike started to get a glance at the game, but was too focused on his own problems to see the big picture”.

Jasmine's reference to “the game”, of course, raised some concerns over possible elements of delusional thinking involved, similarly to what was reportedly Mike's situation when he suffered his mental health issues. However, hearing Jasmine talk and articulate her concepts, we were constantly under the impression that her reality testing was not impacted. Her idea of the “game” resided more in the philosophical realm –– where it served as a metaphor for a rather constraining set of social and family expectations –– rather than being a genuine representation of how Jasmine perceived her concrete reality.

In other words, Jasmine was fully aware that there was no secret agency controlling people through a game, and was referring to it to make her point (“I will not do what people expect of me”).

Ellinor reports that Jasmine has received counselling, since the problem onset, but stopped going as it was, in her words “not clicking with me”.

Previous attempts at solving current problems are a crucial part in the problem deconstruction (19), as they offer a view on the meaning making and family dynamics surrounding the problem itself. Therefore, we were genuinely curious to understand what sense did Jasmine and the family make of the counselling sessions, and what did not “click”.

Apparently, the counsellor shared with Jasmine that part of her anxiety was due to the complicated relationship between her and Ellinor. Specifically, the counsellor hypothesized that Jasmine might have been experiencing a disorganized attachment relationship due to her mom's ongoing depression.

Jasmine's response to this idea was ambivalent. On one hand, she got really angry at the counsellor for blaming her mother and decided to quit the sessions. On the other hand, she often refers to her relationship with Ellinor with terms such as “symbiotic”, “too close” and “constraining”, and often blames her for not being a good role model: “I see her always on the couch and I feel so uninspired…”.

It appears that the blame game that we previously identified as a family pattern involves more agencies and people in position of power and authority. Both the school and the professional counsellors seem to convey a message according to which the parent (and more specifically Ellinor) are to blame for the situation.

This seems to match what observed by Colangelo (20), where some families where the semantic of power is prominent might experience the impact of a social construction of blame, when other agencies or persons with a role of authority are perceived as judgmental. The identified patient, most often a teenager, is likely to react to this process by escalating their behaviour, thus expressing their ambivalence between the loyalty to the parents and the anger towards them.

We are therefore left wondering whether a similar ambivalence is at play here, especially with regards to the relationship between Jasmine and Ellinor.

### 3.4 The lost ambition

As we are keen to explore the influence of the problem, we are curious to understand who Jasmine was, before “the game”.

Ellinor says that Jasmine has always been focused on her academic career, she wanted to be the best, not only for herself, but for women: “she sees all the inequality women go through and always wanted to make a difference”. At school, she has always been involved in groups advocating civil rights and her long term career goal was always to become a lawyer and fight for the civil rights of LGBTQIA+ people.

Josh says that, although his views on human rights did not necessarily align with Jasmine, he used to admire her spirit and determination, and that he struggles to recognize her now: “she has always been very ambitious, a hard worker, never a quitter”.

She always wanted to be the best at everything and last year she pushed them to change her school because the one she attended was not conforming to her standards and ambitions.

Jasmine confirms that the old school had a culture of relationships and everyone was nice, but she wanted to challenge herself. The new school was task oriented and put a lot of pressure on her, but she felt she had to keep going. All this, of course, until she came up with the idea of the “game”.

Josh is also critical of Ellinor, guilty of being too passive towards Jasmine, who perhaps needs a “spur”. He adds, however, that he guiltily left all the responsibility on his wife's shoulders, because he was too busy at work.

Jasmine thinks that her father is a good man, but somehow still prone to a patriarchal view of the world. For instance, he repeatedly stated he could never offer her a job in his company, while he had no issues offering a post to Mike despite his mental health issues and the strained relationship. According to Jasmine, he is also condescending towards Ellinor. She says there is a misogynistic culture in the family and it probably comes from the previous generation. In Josh's family, the roles were strictly assigned, the father being the breadwinner and the mother taking care of the house and the children. Even more blatantly, Jasmine says that Ellinor's family all revolved around the powerful figure of Ellinor's father, “a true tyrant”, whereas Ellinor's mother was submissive, subjugated and suffering from undiagnosed depression.

Josh defends his choice of not thinking about employing Jasmine in the future, saying that he did not make the rules and finance is a male-dominated world. He believes Jasmine would be extremely unhappy as she would have to face daily discrimination, and he wants to protect her.

Ellinor agrees with her husband, and points out that he is the one who pays for Jasmine's expensive school and has always invested in her studies.

However, Jasmine thinks that her mom talks like that because she suffers from internalized misogyny.

### 3.5 The presenting problem, its meaning and function: entering the “as if” territory

As previously clarified, although the whole idea of the “game” clearly might have raised concern over her overall well-being and mental health, we did not observe any clinical sign of a significantly altered reality testing or other indicators that a paranoid ideation might be at play. Therefore, despite the themes being similar, Jasmine does not seem to present with the same condition that Mike presented with in 2019.

Instead, it seems that the whole “game” narrative is triggered by the pressure and anxiety that Jasmine experienced when she moved from a nurturing, but rather non-competitive school environment toward a task-driven and extremely “dog eats dog” one.

Her child psychiatrist, who conducted the initial assessment upon the problem onset, seemed to share the same impression, as he reported that Jasmine was suffering from anxiety and mild depression, not from a psychotic or otherwise reality altering conditions.

This was a very important element of assessment for us, especially since we were entering the territory of the metaphor, where “as if” is a core component. We would have refrained from it if we were to believe there could be a risk for Jasmine to take it literally.

Again, ambition and power seem to be the crucial dimensions at play.

As observed by Ugazio (13), a key issue, when dealing with families where the semantic of power is crucial, is often the ongoing battle to define the relationship. In conversations where the most important thing is who wins and who loses, who is “up” and who is “down”, other important emotions might be suffocated and silenced. Among those, fear (intended as the primal emotional reaction to a threat to survival) is often subjugated by the thrill of the fight and the intoxicating sense of confidence that comes from standing up to power and challenging the authority.

We observe a similar dynamic, for instance, in some cases of anorexia nervosa, where the fear of being judged, of being defeated, of losing, might often overcome the fear of death. This seems the case with Jasmine, *mutatis mutandis*, as she seems to fear the consequences of going to school, and not being a top student, rather than the consequences of quitting and endangering her future.

This becomes clear halfway through session 2, when Jasmine says:

Even if I wanted to go back to school now, it would be too late. I have missed too many classes, I would never be able to reach my goals. I would be at a clear disadvantage when compared to all my peers, it would be a losing battle for me. Better to quit than to be one of the many…

This belief is probably at the core of Jasmine's pattern of return to school. When she goes back on a Friday, she enjoys her peers' company and studying what she loves. However, she also realizes how behind she is and this arguably boosts her anxiety during the weekend, leading to the subsequent withdrawal in a vicious cycle.

Pointing out this aspect of the problem, however, might be strategically dangerous, as it would reinforce a narrative of Jasmine as damaged and it would probably feed into the family belief that Ellinor's poor role modelling is responsible for her daughter's flawed confidence.

Here is when entering the “as if” territory, a metaphorical space where we can explore the “function of the symptom”, might offer a different perspective.

If we were to believe that Jasmine's problem is also a message that she is sending out to her family, an attempt to protect or challenge an aspect of the family life, structure or dynamics, what would this message be? Is it possible to apply the principle of positive connotation (2) to the current situation?

In this respect, we can perhaps hypothesize that it is almost “as if” Jasmine is so loyal to her mother that she feels conflicted about following her own ambitions, thus leaving Ellinor as the only unaccomplished person in the household. “As if” she is reluctant to embrace her father's expectations over her, since this would be an alliance that would leave her mother out. Also, by presenting a problem that Josh cannot solve through his usual, pragmatic means, she is perhaps expressing a need to review the power infrastructure in the family and its patriarchal foundation.

## 4 The intervention: a funeral for the lawyer

Since we devised this hypothesis as a metaphor, we did not rely on a linear, causal correlation between our idea and Jasmine's problem. In other words, at no point we did believe that our hypothesis explained “why” Jasmine developed her self-harming behaviour and school refusal.

Embracing a social constructionist perspective, we were less concerned with how “true” our hypothesis was in terms of linear causation, and more interested in how useful and effective it could be in promoting a shift in the conversation, a transformation in the narrative and ultimately the exploration of different behaviours, both on an individual and an interpersonal level.

By all means, we wanted to find a way to open the “as if” territory to the family.

However, we felt we could not enter such metaphorical space through the linear, constrictive means of explanation. We did not want to explain our hypothesis to the family, we wanted them to experience it.

For this reason, we decided to embed our hypothesis in a suggestion that we shared with the family. While it can be argued that such suggestion recalls the invariable prescription devised by Mara Selvini Palazzoli and Giuliana Prata (8), we moved away from the directive nature of the original version and offered a more collaborative, less expert-positioned translation of its principles.

This seems coherent with the interesting perspective offered by Smith (21), suggesting there could be a 1.5 position for systemic therapists, in between the rigid and directive expert position of first-order cybernetics models and the collaborative, democratic but perhaps not always poignant position held by second-order therapists.

It also connects with Ugazio & Ferrario's (22) proposal of “falsifying experiences” as a way to deliver counterparadoxical interventions in a more collaborative and co-constructed way, still maintaining the strategic spirit of invariable prescriptions, but empowering the client in becoming the protagonist of their own transformation.

We therefore designed a falsifying experience that we named “a funeral for the lawyer”, which delivery is reported below:

It seems to us you have engaged a hopeless battle, where your attempts to get Jasmine back to school clash against her determination to quit the game and her anxiety about a school that seems to be asking too much of her.

This seems to cause more and more suffering every time the hope of Friday afternoons is annihilated on Monday mornings.

Not only it is painful for all of you to experience such distress, it also places you on opposite sides.

We are wondering whether perhaps Jasmine might be trying to tell us that the brilliant lawyer, human rights advocate and women's defender, whom you all nurtured in your minds and hearts, has died. That she didn't survive the test of real life, although she knows, and we know too, that you tried everything to keep her alive.

To us, it looks like Jasmine is determined to kill this promising future lawyer, and no one can prevail over such determination. As you described her, she is brilliant, strong-willed, resolute. There is no way you, or we, for what matters, can prevail over her resolve.

How about, instead of trying so hard to take Jasmine back to school and then blaming yourselves for failing, perhaps we all accept the defeat, and you take some time to mourn this brilliant lawyer together? How would it feel to bury this amazing prospect human rights advocate as a family, reflecting on what you will miss about her, and what she leaves you as a legacy. May you all give her a farewell thought, as she deserves.

Would it be possible for Jasmine, on one of the many afternoons when Ellinor is on the couch, to join her and cry over this loss together? How would it feel for both?

Of course, we understand you might not feel ready to let the lawyer go, as she has been part of the family for so long, so we would respect your decision if you were to decide it's too early, and you want to try again.

But if you decide, as a family, it is time to say farewell to the lawyer that never was, we would like to be here for you and help you in this mourning that we understand to be painful, if you so wish.

Whilst in many respects this “funeral” echoes the counterparadoxical element of invariable prescriptions, our delivery comes in the shape of an experiment, from a stance of curiosity and with a collaborative approach. We make it explicit to the family that what we are suggesting is to engage together in an experiment and, as such, they cannot fail it: if they perform it as suggested, or even if they make creative adjustments of their own, we will have access to a new repertoire of emotions, feelings, thoughts and behaviours that we can discuss in the following sessions. If they don't, we will still be able to co-construct meanings based on what stopped them.

The family listened to our proposal and, while we could clearly read the relief on Ellinor's face and the way a weight seemed to have been removed from her shoulders, we were also conscious of how Josh was not immediately on board of the idea.

Jasmine, sat in between the parents, remained silent and seemed to ponder our words and their implications.

Ellinor asked if we could clarify whether it meant that they had to refrain from taking Jasmine to school even if she asked them too. We answered that it was not our place to make this decision and we trusted them to do what they felt was right. We would have been there to discuss what they did and how it felt, whatever the outcome of their experience.

We agreed to meet in one month to follow up.

According to Ugazio & Ferrario (22), a falsifying experience holds:

(a) an explicit goal, the one that is openly communicated to the client and is aimed at engaging the client in the experience by directly addressing their therapeutic goals and expectations;
(b) an implicit goal, which is somehow hidden strategically in between the lines of the communication, and is at the core of the counterparadoxical intervention.

It is worth noting that both goals can potentially contribute to the change, as both can offer access to new meanings.

With regards to the funeral that we proposed to the family,

(a) The explicit goal was to allow Josh and Ellinor to stop blaming themselves (and each other) for not being able to bring Jasmine back to school, while also acknowledging Jasmine's right for self-determination.
(b) The implicit goal was to remove the element of conflict and challenge between Jasmine and her parents. By offering a way out of the confrontational pattern (Josh and Ellinor want Jasmine back to school, which she resists), we were hoping to also remove the sense of thrill and satisfaction that possibly came to Jasmine by being able to defy her father's authority and assert herself as the one in charge. Hopefully, this could open a space for Jasmine to reflect on her own goals, rather than just keeping herself busy defying her parents', and to put her in touch with deeper feelings and emotions associated with her life choices.

Another aspect in which our proposal differs from the invariable prescriptions, as devised by Selvini Palazzoli and Prata, is that we do not set a desired outcome for the experience, neither explicitly or implicitly. We accept that change can be unpredictable and take the family toward an entirely new direction. In this respect, whilst we hold an expert position in the way we suggest an experiment to the family, we also embrace a not-knowing stance, as we are ready to go with the flow of what the family will bring to the next session and do not expect, or demand, a specific result. This stance, dancing in between knowing and not-knowing, feeds into the idea of a 1.5 position, as described by Smith (21).

## 5 Outcome of the intervention: a (perhaps too) quick farewell

The family attended the following meeting and they seemed eager to discuss what happened in between sessions.

Jasmine says that, after a week of deep thinking and considerations, she decided to go back to school and has been regularly attending since. She mentioned that Ellinor was a bit reluctant at first, because she feared that this would damage her, but she was reassured to see her anxiety lower and they decided “to give it a go”. She says that she is not sure that the current school is the best fit for her, but she would consider changing only after completing the academic year.

No self-harm episode was reported.

Ellinor mentioned that, while her daughter and she spent time on the couch mourning, “as prescribed”, she also had time to think about her future. Since Jasmine is now back to school, she has applied for a part-time job, as “I want to be something more than a depressed empty nester”.

Josh seems happy with the changes, especially with regards to Jasmine. He points out that the family does not need Ellinor to contribute financially, but “I am happy if she is happy”.

He also expresses the desire to close the therapy, “because the problem is now solved” and he considers it dangerous to dig deep when things are going well.

Although agreeing with her husband that they were in a much better place and the original goal for therapy was achieved, Ellinor also expressed concerns about this decision as she felt there could be still room for improvement in family communication and she feared the risk of going back to square one without therapeutic support.

We listened to these concerns and decided to reassure her, and the family, that we believe they were the ones generating the change, so there is no reason to believe this change will fade once we are out of the picture.

Also, we reminded them that they could call us, should they need to protect this change or even if they wanted help with any other matter.

We therefore acknowledged that, although there might be things left unexplored and perhaps not everything is resolved, they did an amazing job as a family and we trusted them to use their strengths to navigate any future challenges.

It was important for us, at this stage, to let the consultation closure to be family-led. We were fully aware that, despite the collaborative delivery of the “funeral” experience, this suggestion was so divergent, from the initial perspective of the family on the problem, that it inevitably created an unbalance of power in the therapeutic relationship between ourselves and the family. Therefore, empowering their decision to close and reassuring them that they were in charge of the change was an attempt to give this power back to them.

## 6 Discussion: a critical evaluation of the intervention

Despite the fast resolution of the presenting problem and some encouraging changes in other members of the family, we acknowledge that a sudden change might also represent a way for the family to escape the pressure represented by the therapy. We could appreciate how painful it was for the family to be subject to the judgment by agencies and professionals with a position of power and authority and, although we tried to convey a non-judgmental perspective, we are aware of the power our position brings. For this reason, it was important to validate and empower their decision to leave, even though there might have been areas left for therapeutic work.

We are also conscious of the power such an intervention carries, and how it places us in a position of authority, despite our efforts to smoother the delivery.

Since its very beginnings, the Milan Approach methods have been subject to criticism and even branded as “dangerous” and “disrespectful” toward the family (23). We do not believe this to be the case, and we consider this criticism as, at least partially, descending from a misinterpretation of the “as if” space where all Milan Approach hypotheses belong.

With that being said, we do not underestimate the possibility that families might feel constrained, having to respond to an intervention where the counterparadox is delivered in the shape of a proposal for change.

We also think that, in family therapy, a collaborative, non-expert positioned approach to families may, in some cases, be the best approach, especially when the family is engaged in co-constructing meanings and there is a strong therapeutic alliance.

This was not the case with Jasmine and her family. Since the beginning, we could sense the concerns and doubts lingering in our conversation. Whilst Ellinor seemed to be keen on having therapy with her family, both Josh and Jasmine, although for different reasons, seemed to have a foot already on the doorstep, ready to leave.

Moreover, a purely conversational approach, based on deconstructing and reauthoring narratives, seemed unable to click with Josh, who was looking for a pragmatic fix to the problem.

We therefore chose a pragmatic approach to encourage the emerging of new meanings, tuning up with a language that seemed predominant in the family.

It might be that, in some situations, the careful, collaborative approach suggested by most family therapists is not welcome by the family. Some families are looking for an expert to tell them how to deal with a problem, or to help them not to worry too much about it.

This is when a 1.5 order approach (21), might be helpful. This is also when the unconventional, inconvenient wisdom of our ancestors might come handy.

Of course, the present paper can be subjected to criticism for being anecdotal, which would be a fair point. Although in our 20 years clinical experience with families, couples and individuals, counteparadoxical interventions in the shape of a falsifying experience (22) have often proven effective in bringing a type 2 change in the family dynamics (4), more high-quality studies would be necessary to ascertain its evidence base. However, we hope that this paper can contribute to a conversation over the underpinning principles and ethics of maintaining the Milan Approach legacy alive.

While we embrace a revolution, as suggested by our era, we should never burn books and destroy monuments, you never know when you may need one.

## Data Availability

The data analyzed in this study is subject to the following licenses/restrictions: The information and data discussed in the paper are derived from three family therapy sessions and are therefore confidential. Anonymized excerpts and redacted information are provided in the paper. Requests to access these datasets should be directed to Ferdinando.Salamino@northampton.ac.uk.
